# Short- and long-term follow-up outcomes of patients with Brucella endocarditis: a systematic review of 207 Brucella endocarditis Cases

**DOI:** 10.1080/21655979.2021.1962683

**Published:** 2021-08-18

**Authors:** Xiufeng Li, Tan Wang, Yuanzhi Wang, Songsong Xie, Wenbo Tan, Ping Li

**Affiliations:** a Department of Nursing, School of Medicine, Shihezi University, Shihezi, China; b Department of Basic Medicine, School of Medicine, Shihezi University, Shihezi, China; c Department of Infectious Diseases, The First Affiliated Hospital of Shihezi University School of Medicine, Shihezi, China

**Keywords:** *Brucella* endocarditis, follow-up outcomes, systematic review, influencing factors

## Abstract

*Brucella* endocarditis is a fatal complication and the most frequent cause of death for human brucellosis. This study aimed to systematically review the literature on the follow-up outcomes of *Brucella* endocarditis and analyze the determinants affecting the follow-up outcomes. The databases PubMed, Web of Science, Embase, and Cochrane were searched using keywords and suitable combinations. All studies reporting the follow-up outcomes of *Brucella* endocarditis were included. Finally, a total of 76 studies (207 patients), including cases or case series, were included. The event rate for patients who underwent short- and long-term follow-up was 12.0% (2 relapsed and 1 died) and 8.1% (6 relapsed and 8 died), respectively. The differences in outcomes between different age groups (18–39, 40–59, and ≥60) were significant (*P* < 0.05, *P* = 0.035). The outcomes of the 18–39 age group were worse than those of the 40–59 age group (OR, 0.277; 95% CI, 0.103–0.748; *P* = 0.011). Accordingly, follow-up (both short- and long-term follow-up) is essential for *Brucella* endocarditis patients, especially for younger patients (18–39 years) in the first 6 months after treatment. The burden of *Brucella* endocarditis related complications were immense. Further studies are needed to explore age-based epidemiology of *Brucella* endocarditis and the exact influencing factors of the follow-up outcomes.

## Introduction

Brucellosis is a zoonotic disease caused by Gram-negative coccobacilli of the genus *Brucella*. The common pathogens of human brucellosis are *B. melitensis, B. abortus, B. suis, B. canis*, and marine species [1]. *B. ceti, B. pinnepedialis, B. microti and B. papionis* are recently isolated species. Besides, there is a group of atypical strains, which are much older in evolutionary terms [2]. The Middle East, Africa, Latin America, Central Asia, and the Mediterranean Basin are endemic areas of brucellosis. Due to the difference between area and career, the high incidence age of brucellosis is different [3]. Males under 45 years of age with the highest risk of brucellosis in Spain [4]. The mean age of Brucellosis patients is 45 years and ages range from 16 to 84 years [5]. Higher brucellosis incidence was reported in males (57.1%) and among the age group 11–20 years (29%) in West Bank of Palestine [6]. Brucellosis incidence in persons aged ≥65 years was more than twice the incidence in persons aged ≤19 years in California [7]. The average incidence of human brucellosis was higher in the 40–65 age group in China [8]. *Brucella* transmission can occur in humans via digestive tract, respiratory tract, and direct contact, such as consuming the unpasteurized milk and/or cheese products, breathing in the bacteria, butchering. After the infection, bacteria travels to the lymph nodes, and then can spread by macrophages to other organs such as spleen, liver, bone marrow and even reproductive organs. The replication of *Brucella* occurs mainly inside the macrophages and non-phagocytic cells, leading to resistance of the human body immune response. Inside the bloodstream, bacteria quickly become intracellular pathogens and makes the use of numerous mechanisms to suppress bactericidal response, causing chronic diseases of brucellosis, such as spondylitis, arthritis, meningitis and endocarditis in infected humans [9]. It is also an occupational disease commonly seen among shepherds, slaughterhouse workers, veterinarians, dairy industry professionals, and microbiology laboratory staff. Brucellosis has a broad spectrum of clinical manifestations and can affect multiple systems of the body, the most common being the osteoarticular system, followed by the genitourinary system [1]. Fatal complications, such as endocarditis (1%) and neurological complications (4%), also occur from time to time [10]. Infectious diseases transmitted from animals to humans is a global threat, which causes a high regional burden [11].

*Brucella* endocarditis is to guide endocarditis caused by *Brucella* infection. *Brucella* endocarditis is rare, but it accounts for 80% of the mortality from brucellosis. The rapid and extensive tissue destruction makes its mortality rate much higher than that caused by other pathogens [12]. Delayed diagnosis or misdiagnosis of other diseases is also associated with high mortality. Isolation of *Brucella* in blood or tissue samples using modified Duke criteria or serological tests is needed in the diagnosis of *Brucella* endocarditis [13]. Infective endocarditis is defined by infection of a native or prosthetic heart valve, the endocardial surface, or an indwelling cardiac device [14]. Transthoracic echocardiography (TTE) should be performed in all cases of suspected infective endocarditis, and trans-esophageal echocardiography (TEE) is performed if initial transthoracic echocardiography images are negative or inadequate in patients suspected of having Infective endocarditis [15]. However, there are no consensus opinion or guideline-recommended standard approach to when to consider surgical intervention remains a challenge in *Brucella* endocarditis [16].

In 1966, a case of severe *Brucella* endocarditis was treated surgically for the first time [17]. Since then, several cases of successful treatment of combined surgical and medical therapy have been reported. With the implementation of surgical treatment, the outcomes of *Brucella* endocarditis have been improved [18]. Despite this, the initial treatment failure rate and relapse rate of brucellosis were higher, which might be related to *Brucella* as an intracellular pathogen [19]. In routine clinical practice, the relapse rate and treatment failure rate within 6 months were about 10% in the multiple-regimen therapy, and even higher than 50% in the single-regimen therapy [20]. Besides, long-term relapses also occur from time to time [21].

However, the specific follow-up outcomes of patients with *Brucella* endocarditis have not yet been systematically reported. This study reviewed all studies in the English language on *Brucella* endocarditis, summarized the current status of follow-up, and analyzed factors affecting the follow-up outcomes. This will also provide a certain direction for improving the prognosis of *Brucella* endocarditis patients.

## Methods

### Search strategy

The databases PubMed, Web of Science, Embase, and Cochrane were comprehensively searched for studies in the English language on *Brucella* endocarditis until 2 December 2019. ‘*Brucella* endocarditis’ and ‘Brucellosis AND Endocarditis’ were used as the keywords for the search.

### Inclusion and exclusion criteria

The inclusion criteria were as follows: studies in English language; studies including patients aged ≥18 years (the guidelines cited in this study are recommended for adult patients); and studies with follow-up information after treatment of *Brucella* endocarditis. The exclusion criteria were as follows: studies in languages other than English, patients aged <18 years, cases of endocarditis were not caused by *Brucella* or caused by more than one organism, patients over the age of 18 who have cardiovascular disease or have a history of certain substrate diseases, patients who lost to follow up, reviews, and other studies not about *Brucella* endocarditis.

### Data extraction

Age, sex, country, valve type (native valve and prosthetic or bioprosthetic valve), valve involvement (aortic, mitral, aortic and mitral, and tricuspid), blood culture, isolate strains, treatment method (medical only or combined medical and surgical), heart failure, infection risk factors, underlying heart disease, and complications were extracted to analyze the factors affecting the follow-up outcomes. Other tests such as serology and echocardiography were also extracted. Follow-up is a method of regular observation of the patient’s condition and the patient’s recovery. It is mainly for patients with mild or stable conditions that do not require hospitalization. The outcomes included cure, relapse, and death. For ease of analysis, in cases reporting the long-term outcome with different time intervals, the longer duration of time was employed in the analysis. As relapses most frequently occurred within 6 months of the initial infection, and 2016 American Association for Thoracic Surgery (AATS) guidelines proposed that patients with infective endocarditis should be followed up for 6 months after operation to verify whether the pathogens were eradicated [22], this study was divided into the short-term follow-up (≤6 months) group and the long-term follow-up (>6 months) group based on the duration of the follow-up.

### Statistical analysis

The Mann–Whitney test was used to compare whether differences existed in patient follow-up outcomes between different age groups (18–39, 40–59, and ≥60 years), involved valves, valve types, treatment methods, blood culture results, isolated strains, heart failure, and sex. The data did not satisfy the parallel test of ordinal logistic regression because of the small sample size (*P* < 0.1). Therefore, relapse and death were combined as one dependent variable, and binary logistic regression was performed to analyze the relationships between follow-up outcomes and age groups, valve types, blood culture results, involved valves, heart failure, and follow-up time. All statistical operations were completed using IBM SPSS Statistics 20.0. A *P* value <0.05 was considered statistically significant.

## Results

### *Identification, screening, and inclusion of* Brucella *endocarditis cases*

**Figure 1.**
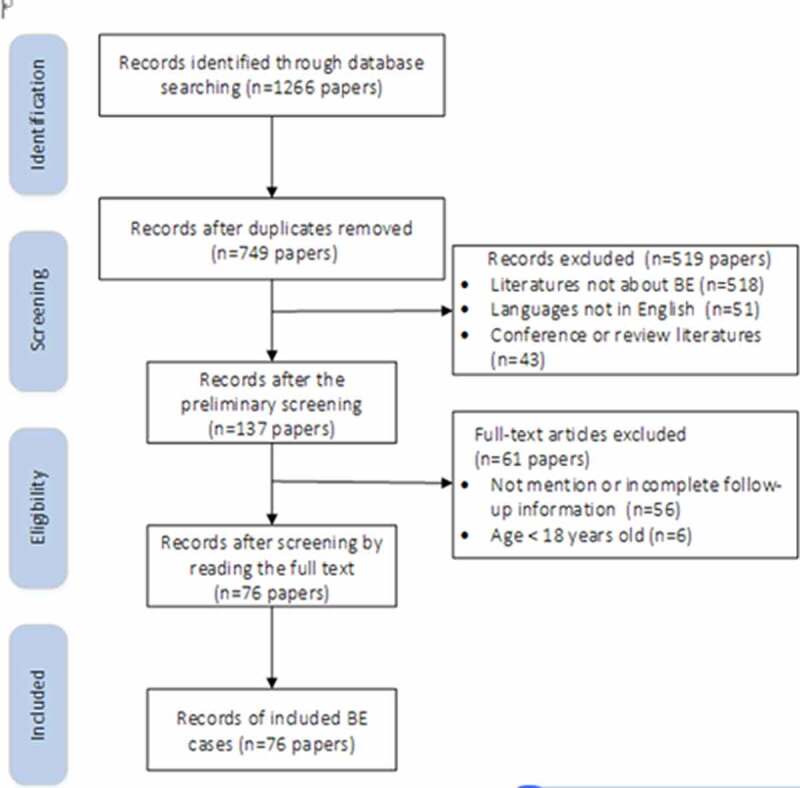
Study selection flow diagram

**Figure 2.**
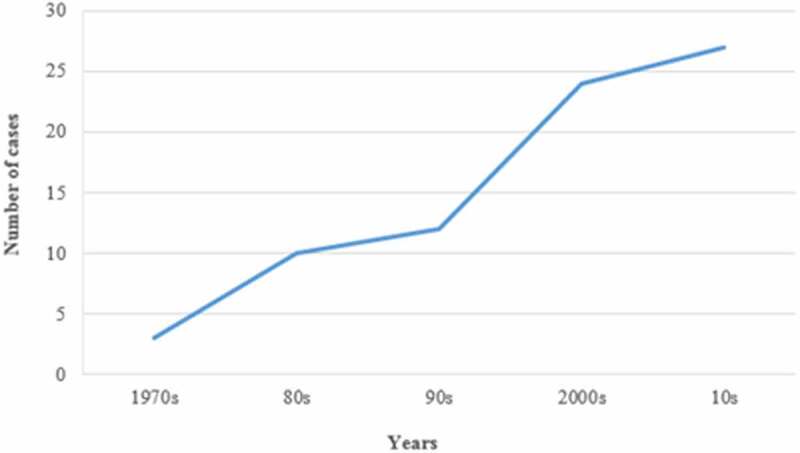
The trend of publication distribution between 1970 and 2019

The identification and screening are summarized in Figure 1. After screening by abstract and full text, finally 76 studies (online Supplementary Table S1) were included (between 1970 and 2019), with 207 cases. The trend of publication distribution (1970–2019) is shown in Figure 2.

BE, *Brucella* endocarditis

### Demographic features of patients

**Figure 3.**
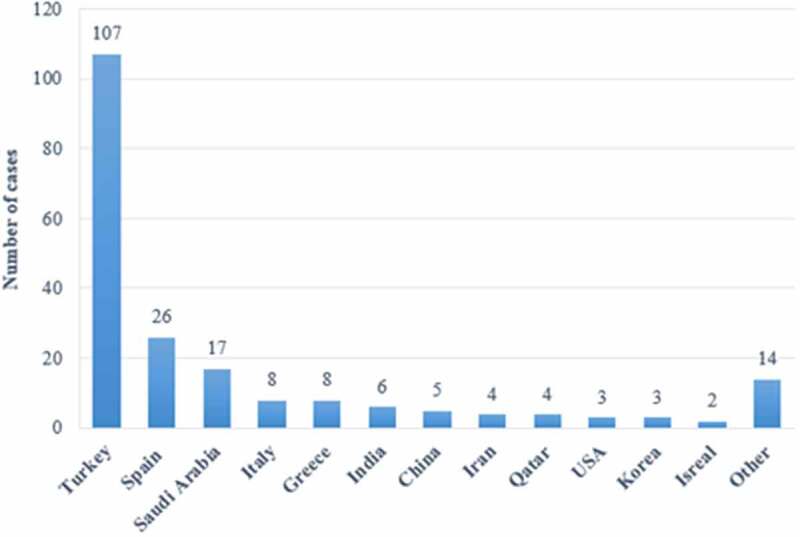
The country-based distribution of patients with *Brucella* endocarditis

A total of 207 patients (74.9% male and 25.1% female; Table 1) were included in this study, with a median age of 45 years (Q1–Q3 35–52) (range 18–82 years). More than half of the cases (51.7%) were from Turkey, followed by Spain (12.6%). The trend of distribution based on specific countries is shown in Figure 3. The main route of disease transmission was through consumption of unpasteurized dairy (34.8%, 72 patients), direct animal contact (24.6%, 51 patients), or both (4.3%, 9 patients); the transmission information for the rest (36.3%, 75 patients) was not mentioned (Table 1). Two patients were infected through foreign travel (Turkey [23] and Greece [24]).

**Table 1.** The basic information of *Brucella* endocarditis patients

Parameters	Value/n	Percentage(%)
Median age(years)(n = 207)	45(35–52)	
Sex(n = 207) Male Female	155 52	74.9 25.1
Risk factors(n = 132) Unpasteurized dairy consumption Animal contact only Unpasteurized dairy + Animal contact	72 51 9	34.8 24.6 4.3
Underlying cardiac condition(n = 145) Aortic valve insufficiency Rheumatic heart disease Aortic stenosis Bicuspid aortic valve Prosthetic valve Mitral valve insufficiency Mitral stenosis Pacemaker placement	45 36 12 10 10 8 6 5	70.0 21.7 17.4 5.8 4.8 4.8 3.9 2.9 2.4
Treatment(n = 205) Combined surgical and medical therapy Medical therapy only	179 26	86.5 12.6
Blood culture (n = 176) Positive Negative	114 62	55.1 30.0
Infecting strain(n = 134) *B. melitensis* *B. abortus* *B. suis* *B. canis*	120 10 3 1	58.0 4.8 1.4 0.5
Valve style(n = 201) Native valve Prosthetic valve	171 30	82.6 14.5
Involved valve(n = 205) AV MV AV + MV Pacemaker AV + MV + TV ASD TV Laboratory examination ESR↑ CRP↑ Titer(n = 164) <1:320 ≥1:320 ≥1:640 ≥1:1280 Vegetations ≥10 mm	120 41 35 4 1 3 1 123 59 16 78 24 46 163 30	58.0 19.8 16.9 2.0 0.5 1.4 0.5 59.4 28.5 7.7 37.7 11.6 22.2 78.7 14.5
Complications(n = 97) Abscess Embolic event Renal complication Pulmonary complications Arrhythmia Spondylitis Paravalvular leak Neurobrucellosis Other Heart failure Event rate (n = 17) ≤6 months >6 months	28 13 12 9 5 5 4 2 19 115 3 14	46.9 13.5 6.2 5.8 4.3 2.4 2.4 1.9 1.0 9.2 55.6 11.1 8.2

*AV* aortic valve, *_BV_* mitral valve, *TV* Tricuspid valve, *ASD* Atrial Septal Defect, *ESR* erythrocyte sedimentation rate, *CRP* C-reactive protein

### *Clinical characteristics of* Brucella *endocarditis patients*

Among the 207 patients, 87.0% (180 patients) were cured, 9.2% (19 patients) died, and 3.9% (8 patients) relapsed. The information on relapsed and dead patients is shown in online Supplementary Table S2 and online Supplementary Table S3, respectively. A total of 82.6% (171 patients) of the affected valves were native valves, and only 14.5% (30 patients) were prosthetic valves (Table 1). The aortic valve (58.0%, 120 patients) was the most frequently involved, mitral valve (19.8%, 41 patients) was the second, and both were involved in 16.9% of patients (35). In this study, five patients had pacemaker infections, and one had simultaneous involvement of the tricuspid valve (Table 1). *Brucella* endocarditis appeared in the valves with or without basic lesions. Moreover, 70.0% (145 patients) had underlying cardiac lesions; aortic valve insufficiency (21.7%, 45 patients) and rheumatic heart disease (17.4%, 36 patients) were the main types of lesions (Table 1). Moreover, 46.9% (97 patients) showed complications. The most common complications were abscesses (13.5%, 28 patients) and embolism (6.2%, 13 patients). Further, 55.6% (115 patients) had heart failure, 86.5% (179 patients) of the patients received a combined surgical and medical treatment, and only 12.6% (26 patients) of the patients received medical treatment only (Table 1).

The blood culture results showed that 55.1% (114 patients) of the patients were positive, 30.0% (62 patients) of the patients were negative, and more than half of the patients (58.0%, 120 patients) were infected with *B. melitensis*. Further, the number of patients infected with *B. abortus* and *B. suis* was 10 and 3, respectively, and only 1 patient was infected with *B. canis* (Table 1). Laboratory examination showed that 59.4% (123 patients) had an elevated erythrocyte sedimentation rate, and 28.5% (59 patients) had elevated C-reactive protein level (Table 1). Echocardiography showed that 78.7% (163 patients) had vegetation, and 14.5% (30 patients) had vegetation greater than 10 mm. The serological titer of patients is shown in Table 1.

### Univariate analysis results

Except for 10 patients who died before and after the treatment, all patients were followed up after the treatment. Again, 13.1% (27 patients) of patients were followed up for less than 6 months, and 82.1% (170 patients) of patients for more than 6 months; the median follow-up time was 24 months (Q1–Q3 12–47). Significant differences in follow-up outcomes were found between different age groups (*P* = 0.035). No significant differences in follow-up outcomes were found between other factors (all *P* > 0.05) (online Supplementary Table S4).

### Multivariate analysis results

Multivariate analysis showed that age was an independent factor for follow-up outcomes. The outcomes of patients aged between 18 and 39 years were worse than those of patients aged between 40 and 59 years (OR, 0.277; 95% CI, 0.103–0.748, *P* = 0.011). Other possible factors did not influence the follow-up outcomes (all *P* > 0.05) (Table 2).

**Table 2.** Multivariate Binary logistic regression analysis of influencing factors for follow-up outcomes

Indicators	Odds Ratio	P value	95% CI
Age 40–59 Age ≥ 60	0.277 0.323	0.011 0.165	0.103–0.748 0.065–1.594
Medical treatment	0.404	0.404	0.048–3.400
Heart failure Prosthetic valve	2.467 0.277	0.104 0.232	0.830–7.331 0.034–2.273
AV involved MV involved	0.479 0.407	0.225 0.274	0.145–1.575 0.081–2.038

*AV* aortic valve, *BV* mitral valve

## Discussion

Brucellosis is still an important public health problem worldwide. The annual incidence rates per million population in some endemic regions are as follows: Saudi Arabia (214.4), Iran (238.6), Turkey (262.2), Iraq (278.4), and Syria (1603.4). However, according to the World Health Organization, the real incidence is 10–25 times more than what has been reported [25].

The endocardial lesions of brucellosis are the result of direct invasion of the endocardium by the organisms. Lesions resembling Aschoff bodies are commonly presented in *Brucella* endocarditis. Even without the ‘typical’ Aschoff bodies, it is not uncommon to find a local aggregation of lymphocytes and mononuclear cells as well as patchy areas of interstitial fibrosis [12]. However, Osler node on fingertips is often encountered in infective endocarditis [15]. Besides, anti-*Brucella* IgG was the most robust biomarker of complicated brucellosis [26].

### *Follow-up outcomes of* Brucella *endocarditis*

This perhaps is the first systematic review of the follow-up outcomes of patients with *Brucella* endocarditis. In this systematic review, a higher proportion of event rate was found in the short-term follow-up of patients with *Brucella* endocarditis.

According to 2016 AATS guidelines, the risk of relapse existed during the first 6 months after the surgery. Therefore, patients should be followed up by an infectious disease specialist in the first 6 months [22]. Besides, abscess formation and complete valve damage might be related to poor outcomes [27]. In the present study, 86.5% (179) of the patients received surgical treatment, 70.0% (145) of the patients had an underlying cardiac lesion, and 19.7% (41) of the patients had abscess formation or embolic event, which might be related to poor outcomes of the patients with the short-term follow-up.

After 6 months, a continued follow-up with a cardiologist is more appropriate. Patients with *Brucella* endocarditis receive bioprosthetic valves or an allograft with a limited durability; therefore, a careful long-term follow-up with a cardiologist is important [22]. Sannikova’s study found that continuous management and follow-up helped reduce medical and social losses caused by brucellosis infection [28].

### *Age and* Brucella *endocarditis follow-up outcomes*

Compared with age between 18 and 39 years, age between 40 and 59 years was a protective factor for *Brucella* endocarditis (OR, 0.277; 95% CI, 0.103–0.748; *P* = 0.011), contrary to the reports that old age was a high-risk factor for poor prognosis of infective endocarditis [14]. This indicates that in the case of the same disease, the follow-up frequency of young people should be strengthened and the follow-up time should be appropriately extended. The reasons might be as follows: the ages of onset between *Brucella* endocarditis and infective endocarditis were different. The median age of included patients in this study was 45 years (Q1–Q3 35–52), which was lower than that in the reported study about infective endocarditis [29]. Moreover, 87.4% (181) of the patients were younger than 60 years, only 12.6% (26) of the patients were older than 60 years (the average age of infective endocarditis) [29]. About half of the cases were found in Turkey, and the incidence of brucellosis in Turkey was the highest in younger patients (54% aged between 14 and 34 years), perhaps reflecting the traditional role of children in raising livestock [30]. However, patients with infective endocarditis were old and sick, often accompanied by multiple comorbidities [14].

Moreover, previous studies showed that the medical adherence of older patients was higher than that of younger patients [31–33]. The facts also proved that the chronic rate of brucellosis in people with low treatment compliance was higher than those with good compliance [28]. In addition, drug abuse was associated with an increased risk of relapse [22]. 33–35 years was the peak age for drug abuse in both sexes [34], which is just between 18 and 39 years. Coincidentally, one patient was a drug abuser in the present study [35]. Age-specific epidemiology and trends of infective endocarditis were, however, inadequately known [29], not to mention *Brucella* endocarditis. Therefore, the role of drugs in the disease outcomes needs to be verified through further studies.

### Other possible influencing factors

Keshtkar-Jahromi [18] found that surgery decreased mortality from 32.7% in the medical treatment–only group to 6.7% in the combined surgical and medical treatment group. However, the influence of surgical treatment on the outcomes of *Brucella* endocarditis was not found in this study because of the small number of patients. The reason might be that *Brucella* endocarditis was more likely to cause fibrosis, hyalinization, and calcification than endocarditis caused by other bacteria. Most patients died of valve deformity and congestive heart failure because of the aforementioned reasons, which were hard to treat with medicine only [12].

Control of infection with preoperative antibiotic therapy and immediate surgery after clinical stabilization has also been recommended. The most commonly used antimicrobial regimen consisted of rifampin, tetracycline, and an aminoglycoside or cotrimoxazole. However, the choice of antibiotics, antibiotic regimens, or the length of treatment with antibiotics did not show any significant effect on patient outcomes [18].

The presence of prosthetic valve endocarditis did not significantly increase mortality or relapse rate compared with native valves, which was in line with the study by Keshtkar-Jahromi [18]. This study reported that patients with signs and symptoms of congestive heart failure had higher mortality, which might be because of the small sample size (207 patients) compared with the other studies (308 patients). Aortic valve infective endocarditis was more invasive compared with mitral valve infective endocarditis (true for both native valve endocarditis and prosthetic valve endocarditis). Despite this, the outcomes were worse after the surgical treatment with mitral valve infective endocarditis compared with aortic valve infective endocarditis [36]. However, no difference in patient outcomes between mitral valve *Brucella* endocarditis and aortic valve *Brucella* endocarditis was found.

In the present study, the number of publications on *Brucella* endocarditis in Turkey was high. The possible reasons were as follows: First, isolate strains might be the cause. As a fatal complication of human brucellosis, *Brucella* endocarditis was rare in Western countries where the infective agent was *B. abortus*, which led to moderate disease and uncommon supportive and disabling complications. However, *B. melitensis* led to more severe disease and disabling symptoms [13]. *B. melitensis* probably accounted for most cases of human brucellosis in Turkey, with *B. abortus* being the second most frequent. Besides, patients were generally of low socioeconomic class and educational level [30]. In addition, the number of publications of human brucellosis found in Turkey was high [37].

The trend of the number of cases of brucellosis in Turkey was similar to that of publication distribution of *Brucella* endocarditis in this study. Cases of human brucellosis were rarely registered in Turkey before the 1980s. During the period 1980–2005, 189,226 cases of human brucellosis were reported officially. About 90,000 of these were registered between 2000 and 2005 (approximately 15,000 cases/year) [30]. However, this trend might be related to the improvement in diagnostic technology and sanitation level.

### Relapses and prevention

The goal of antibrucellar treatment is to alleviate and shorten the symptomatic period and reduce complications, relapses, and chronicity. Contemporary trends in the treatment of human brucellosis are postulated on the ability of *Brucella* to persist in host macrophages through the inhibition of phagolysosome fusion and to survive for prolonged periods intracellularly without restricting basic cellular functions. As a result of this and despite satisfactory antibiotic treatment, relapses and therapeutical failures are inevitable to a certain degree. The current principles for the treatment of brucellosis advocate for a long enough treatment duration combined with antimicrobial regimens that possess activity in the intracellular acidic environment [38].

Aminoglycosides (gentamicin or streptomycin with doxycycline) are associated with lower rate of relapse in brucellosis [39]. Medical therapy may play an important role solely in the management of *Brucella* endocarditis patients with high re-operative risks [40]. Various measures to control zoonotic brucellosis are the only way to eradicate the disease as man is a dead end host [41], such as vaccinate animals to prevent the spread of disease to humans.

## Limitations

This study had the following limitations. First, this study was a retrospective study, the included studies were published case reports or case series, and the information on some cases was not very comprehensive, especially case series. Second, this study included only documents in English; the documents in other languages, such as French and Spanish, were excluded. Besides, there are no data about treatment details. Finally, the sample size was not large enough (207 patients). Further, larger sample studies or controlled trials are required to validate the results of this study.

## Conclusions

In conclusion, follow-up (both short- and long-term follow-up) is essential for *Brucella* endocarditis patients. Younger patients (18–39 years) should be followed up more frequently, especially in the first 6 months after treatment. Besides, the burden of *Brucella* endocarditis complications were higher among *Brucella* endocarditis patients, clinicians should be alert to the presence of *Brucella* endocarditis, especially in non-endemic areas and in younger patients (18–39 years). In addition, Turkey is the most endemic region for *Brucella* endocarditis. More cases or well-designed studies are required to explore age-based epidemiology of *Brucella* endocarditis and the exact influencing factors of the follow-up outcomes, as these may provide insight into prevention and management of the disease.

## Supplementary Material

Supplemental Material

## Highlights

1. The high incidence age of human brucellosis varies by region and occupation.
2. Follow-up outcomes of younger *Brucella* endocarditis patients may be worse than older patients.
3. There are no differences between short- and long-term follow-up outcomes of *Brucella* endocarditis patients.
