# Supplementary material for: Short- and long-term follow-up outcomes of patients with Brucella endocarditis: a systematic review of 207 Brucella endocarditis Cases
Source: Bioengineered. 2021 Aug 18;12(1):5162–72. doi: 10.1080/21655979.2021.1962683 (PMC8806701; doi:10.1080/21655979.2021.1962683)
Supplement: Supplemental Material [file KBIE_A_1962683_SM9446.zip › supplementary/online Supplementary Table 1.docx]

**Table 1** The basic information of *Brucella* endocarditis patients

| **Parameters** | **Value/n** | | **Percentage(%)** |  |
| --- | --- | --- | --- | --- |
| Median age(years)(n=207) | 45(35-52) | |  |  |
| Sex（n=207）  Male  Female | 155  52 | | 74.9  25.1 |  |
| Risk factors(n=132)  Unpasteurized dairy consumption  Animal contact only  Unpasteurized dairy + Animal contact | 72  51  9 | | 34.8  24.6  4.3 |  |
| Underlying cardiac condition(n=145)  Aortic valve insufficiency  Rheumatic heart disease  Aortic stenosis  Bicuspid aortic valve  Prosthetic valve  Mitral valve insufficiency  Mitral stenosis  Pacemaker placement | 45  36  12  10  10  8  6  5 | | 70.0  21.7  17.4  5.8  4.8  4.8  3.9  2.9  2.4 |  |
| Treatment(n=205)  Combined surgical and medical therapy  Medical therapy only | | 179  26 | 86.5  12.6 | |
| Blood culture (n=176)  Positive  Negative | | 114  62 | 55.1  30.0 | |
| Infecting strain(n=134)  *B. melitensis*  *B. abortus*  *B. suis*  *B. canis* | | 120  10  3  1 | 58.0  4.8  1.4  0.5 | |
| Valve style(n=201)  Native valve  Prosthetic valve | | 171  30 | 82.6  14.5 | |
| Involved valve(n=205)  AV  MV  AV + MV  Pacemaker  AV + MV + TV  ASD  TV  Laboratory examination  ESR↑  CRP↑  Titer（n=164）  <1:320  ≥1:320  ≥1:640  ≥1:1280  Vegetations  ≥10mm | | 120  41  35  4  1  3  1  123  59  16  78  24  46  163  30 | 58.0  19.8  16.9  2.0  0.5  1.4  0.5  59.4  28.5  7.7  37.7  11.6  22.2  78.7  14.5 | |
| Complications(n=97)  Abscess  Embolic event  Renal complication  Pulmonary complications  Arrhythmia  Spondylitis  Paravalvular leak  Neurobrucellosis  Other  Heart failure  Event rate (n=17)  ≤6 months  >6 months | | 28  13  12  9  5  5  4  2  19  115  3  14 | 46.9  13.5  6.2  5.8  4.3  2.4  2.4  1.9  1.0  9.2  55.6  11.1  8.2 | |

*AV* aortic valve, *_BV_* mitral valve, *TV* Tricuspid valve, *ASD* Atrial Septal Defect, *ESR* erythrocyte sedimentation rate, *CRP* C-reactive protein
