# Supplementary material for: Short- and long-term follow-up outcomes of patients with Brucella endocarditis: a systematic review of 207 Brucella endocarditis Cases
Source: Bioengineered. 2021 Aug 18;12(1):5162–72. doi: 10.1080/21655979.2021.1962683 (PMC8806701; doi:10.1080/21655979.2021.1962683)
Supplement: Supplemental Material [file KBIE_A_1962683_SM9446.zip › supplementary/online Supplementary Table 2.docx]

**Table 2** Multivariate Binary logistic regression analysis of influencing factors for follow-up outcomes

| **Indicators** | **Odds Ratio** | **P value** | **95% CI** |
| --- | --- | --- | --- |
| Age 40-59  Age ≥ 60 | 0.277  0.323 | 0.011  0.165 | 0.103-0.748  0.065-1.594 |
| Medical treatment | 0.404 | 0.404 | 0.048-3.400 |
| Heart failure  Prosthetic valve | 2.467  0.277 | 0.104  0.232 | 0.830-7.331  0.034-2.273 |
| AV involved  MV involved | 0.479  0.407 | 0.225  0.274 | 0.145-1.575  0.081-2.038 |

*AV* aortic valve, *BV* mitral valve
