# Supplementary material for: Short- and long-term follow-up outcomes of patients with Brucella endocarditis: a systematic review of 207 Brucella endocarditis Cases
Source: Bioengineered. 2021 Aug 18;12(1):5162–72. doi: 10.1080/21655979.2021.1962683 (PMC8806701; doi:10.1080/21655979.2021.1962683)
Supplement: Supplemental Material [file KBIE_A_1962683_SM9446.zip › supplementary/online Supplementary Table 3.docx]

**Online Supplementary Table 3** The information of 19 died patients

| **No** | **Year** | **Age(years)** | **Sex** | **Treatment** | **Died time** | **Cause** |
| --- | --- | --- | --- | --- | --- | --- |
| 1 | 1990 | 25 | M | Died before treatment. | 25h after admission | Fatal cardiac arrest |
| 2 | 1989 | 30 | M | Died before surgery treatment. | - | - |
| 3 | 2012 | 35 | M | Died before treatment. | 5th day of admittance | Developed respiratory and cardiac arrest |
| 4 | 1970 | 23 | M | Surgery plus medication | 38th day after operation | Sudden cardiac arrest. |
| 5 | 2004 | 34 | M | Surgery plus medication |  | Renal failure |
| 6 | 2006 | 53 | M | Medical therapy only | 1 month | Refused further treatment |
| 7 | 1998 | 65 | M | Surgery plus medication | 24 months | Massive cerebrovascular accident |
| 8 | 1999 | 50 | M | Surgery plus medication | 144 months | 4th redo AVR |
| 9 | 2005 | 42 | F | Surgery plus medication | 124 months | Severe stroke |
| 10 | 2009 | 29 | M | Surgery plus medication | 180 months | Severe stroke |
| 11 | 2007 | 38 | F | Surgery plus medication | 15 days after operation | - |
| 12 | 2011 | 29 | M | Surgery plus medication | 184 months | Congestive heart failure |
| 13 | 2011 | 42 | F | Surgery plus medication | 124 months | Embolic stroke |
| 14 | 2016 | 18 | M | Surgery plus medication | Soon after operation | - |
| 15 | 1988 | 25 | F | Surgery plus medication | 12 months | Unknown cause |
| 16 | 2000 | 22 | F | Surgery plus medication | Died in hospital | Stroke |
| 17 | 2003 | 45 | M | Surgery plus medication | Died in hospital | Prolonged intubation and pneumonia. |
| 18 | 1996 | 35 | M | Surgery plus medication | Soon after operation | - |
| 19 | 1998 | 39 | M | Surgery plus medication | 42 months | Left ventricular failure |

*M* Male, *F* Female
