# Supplementary material for: Short- and long-term follow-up outcomes of patients with Brucella endocarditis: a systematic review of 207 Brucella endocarditis Cases
Source: Bioengineered. 2021 Aug 18;12(1):5162–72. doi: 10.1080/21655979.2021.1962683 (PMC8806701; doi:10.1080/21655979.2021.1962683)
Supplement: Supplemental Material [file KBIE_A_1962683_SM9446.zip › supplementary/online Supplementary Table 4.docx]

**Supplementary Table 4** Univariate analysis of influencing factors for *Brucella* endocarditis outcomes

| **Variables** | **Frequency(n)** | **Z value^a^** | **P value** |
| --- | --- | --- | --- |
|  | **Death Relapse Cure** |  |  |
| Follow-up  ≤6 moths  >6 months  Age  18-39  40-59  ≥60  Treatment | 1 2 24  8 6 156  13 4 64  5 4 91  1 1 24 | -1.091  6.723^b^  -0.706 | 0.275  0.035  0.448 |
| Combined therapy  Medical therapy  Valve type  Aortic valve  Mitral valve  Other  Valve type | 15 9 155  2 0 24  9 7 104  4 0 37  5 2 37 | 0.624^b^  -0.594 | 0.732  0.552 |
| Native valve  Prosthetic valve  Blood culture | 16 9 146  3 0 27 | -0.658 | 0.510 |
| Positive  Negative  Gender | 14 4 96  3 5 54 | -0.424 | 0.672 |
| Male  Female | 14 6 135  5 3 44 |  |  |
| Isolated strain  *B. melitensis*  Other  Heart failure  Yes  No  Complications  Yes  No  Basic cardiac lesions  Yes  No | 1. 6 102 2. 0 13 3. 8 98   9 1 81  11 6 80  8 3 99    16 5 124  3 4 55 | -0.757  -0.671  -1.543    -0.717 | 0.449  0.502  0.123  0.474 |

Combined therapy: Combined surgical and medical therapy.

^a^ Z value was derived from a Mann-Whitney Test.

^b^ Chi-Square value was derived from a Kruskal Wallis Test.
